# Supplementary material for: Elicitation of Potent Neutralizing Antibody Responses by Designed Protein Nanoparticle Vaccines for SARS-CoV-2
Source: Cell. 2020 Nov 25;183(5):1367–1382.e17. doi: 10.1016/j.cell.2020.10.043 (PMC7604136; doi:10.1016/j.cell.2020.10.043)
Supplement: Data S1. Amino Acid Sequences of Novel Proteins Used in This Study, Related to Figures 1 and 2 [file mmc1.pdf]

Cell, Volume *183*

Supplemental Information

Elicitation of Potent Neutralizing Antibody Responses by Designed Protein Nanoparticle Vaccines for SARS-CoV-2

Alexandra C. Walls, Brooke Fiala, Alexandra Schäfer, Samuel Wrenn, Minh N. Pham, Michael Murphy, Longping V. Tse, Laila Shehata, Megan A. O'Connor, Chengbo Chen, Mary Jane Navarro, Marcos C. Miranda, Deleah Pettie, Rashmi Ravichandran, John C. Kraft, Cassandra Ogohara, Anne Palser, Sara Chalk, E-Chiang Lee, Kathryn Guerriero, Elizabeth Kepl, Cameron M. Chow, Claire Sydeman, Edgar A. Hodge, Brieann Brown, Jim T. Fuller, Kenneth H. Dinnon III, Lisa E. Gralinski, Sarah R. Leist, Kendra L. Gully, Thomas B. Lewis, Miklos Guttman, Helen Y. Chu, Kelly K. Lee, Deborah H. Fuller, Ralph S. Baric, Paul Kellam, Lauren Carter, Marion Pepper, Timothy P. Sheahan, David Veesler, and Neil P. King

Table S1. Amino Acid Sequences of Proteins Used in This Work, Related to Figures 1-6

>RBD-8GS-I53-50A
MGILPSPGMPALLSLVSLLSVLLMGCVAETGTRFPNITNLCPFGEVFNATRFASVYAWNRKRISNCVADYSVLYNSASFS
TFKCYGVSPTKLNDLCFTNVYADSFVIRGDEVRQIAPGQTGKIADYNYKLPDDFTGCVIAWNSNNLDSKVGGNYNYLYR
LFRKSNLKPFERDISTEIYQAGSTPCNGVEGFNCYFPLQSYGFQPTNGVGYQPYRVVVLSFELLHAPATVCGPKKSTGG
SGGSGSEKAAKAEEAARKMEELFKKHKIVAVLRANSVEEAIEKAVAVFAGGVHLIEITFTVPDADTVIKALSVLKEKGAIIG
AGTVTSVEQARKAVESGAEFIVSPHLDEEISQFAKEKGVFYMPGVMTPTELVKAMKLGHTILKLFPGEVVGPQFVKAMK
GPFPNVKFVPTGGVNLDNVAEWFKAGVLAVGVGSALVKGTPDEVREKAKAFVEKIRGATEGGSHHHHHHHH

>RBD-12GS-I53-50A
MGILPSPGMPALLSLVSLLSVLLMGCVAETGTRFPNITNLCPFGEVFNATRFASVYAWNRKRISNCVADYSVLYNSASFS
TFKCYGVSPTKLNDLCFTNVYADSFVIRGDEVRQIAPGQTGKIADYNYKLPDDFTGCVIAWNSNNLDSKVGGNYNYLYR
LFRKSNLKPFERDISTEIYQAGSTPCNGVEGFNCYFPLQSYGFQPTNGVGYQPYRVVVLSFELLHAPATVCGPKKSTGS
GSGGSGGSGSEKAAKAEEAARKMEELFKKHKIVAVLRANSVEEAIEKAVAVFAGGVHLIEITFTVPDADTVIKALSVLKEK
GAIIGAGTVTSVEQARKAVESGAEFIVSPHLDEEISQFAKEKGVFYMPGVMTPTELVKAMKLGHTILKLFPGEVVGPQFV
KAMKGPFPNVKFVPTGGVNLDNVAEWFKAGVLAVGVGSALVKGTPDEVREKAKAFVEKIRGATEGGSHHHHHHHH

>RBD-16GS-I53-50A
MGILPSPGMPALLSLVSLLSVLLMGCVAETGTRFPNITNLCPFGEVFNATRFASVYAWNRKRISNCVADYSVLYNSASFS
TFKCYGVSPTKLNDLCFTNVYADSFVIRGDEVRQIAPGQTGKIADYNYKLPDDFTGCVIAWNSNNLDSKVGGNYNYLYR
LFRKSNLKPFERDISTEIYQAGSTPCNGVEGFNCYFPLQSYGFQPTNGVGYQPYRVVVLSFELLHAPATVCGPKKSTGG
SGGSGSGGSGGSGSEKAAKAEEAARKMEELFKKHKIVAVLRANSVEEAIEKAVAVFAGGVHLIEITFTVPDADTVIKALS
VLKEKGAIIGAGTVTSVEQARKAVESGAEFIVSPHLDEEISQFAKEKGVFYMPGVMTPTELVKAMKLGHTILKLFPGEVV
GPQFVKAMKGPFPNVKFVPTGGVNLDNVAEWFKAGVLAVGVGSALVKGTPDEVREKAKAFVEKIRGATEGGSHHHHH
HHH

>I53-50B.4PT1
MNQHSHKDHETVRIAVVRARWHAEIVDACVSAFEAAMRDIGGDRFAVDVFDVPGAYEIPLHARTLAETGRYGAVLGTA
FVVNGGIYRHEFVASAVINGMMNVQLNTGVPVLSAVLTPHNYDKSKAHTLLFLALFAVKGMEAARACVEILAAREKIAA

>Monomeric SARS-CoV-2 RBD
MGILPSPGMPALLSLVSLLSVLLMGCVAETGTRFPNITNLCPFGEVFNATRFASVYAWNRKRISNCVADYSVLYNSASFS
TFKCYGVSPTKLNDLCFTNVYADSFVIRGDEVRQIAPGQTGKIADYNYKLPDDFTGCVIAWNSNNLDSKVGGNYNYLYR
LFRKSNLKPFERDISTEIYQAGSTPCNGVEGFNCYFPLQSYGFQPTNGVGYQPYRVVVLSFELLHAPATVCGPKKSTHH
HHHHHH

>SARS-CoV-2 S-2P Trimer
MGILPSPGMPALLSLVSLLSVLLMGCVAETGTQCVNLTTRTQLPPAYTNSFTRGVYYPDKVFRSSVLHSTQDLFLPFFSN
VTWFHAIHVSGTNGTKRFDNPVLPFNDGVYFASTEKSNIIRGWIFGTTLDSKTQSLLIVNNATNVVIKVCEFQFCNDPFLG
VYYHKNNKSWMESEFRVYSSANNCTFEYVSQPFLMDLEGKQGNFKNLREFVFKNIDGYFKIYSKHTPINLVRDLPQGFS
ALEPLVDLPIGINITRFQTLLALHRSYLTPGDSSSGWTAGAAAYYVGYLQPRTFLLKYNENGTITDAVDCALDPLSETKCT
LKSFTVEKGIYQTSNFRVQPTESIVRFPNITNLCPFGEVFNATRFASVYAWNRKRISNCVADYSVLYNSASFSTFKCYGV
SPTKLNDLCFTNVYADSFVIRGDEVRQIAPGQTGKIADYNYKLPDDFTGCVIAWNSNNLDSKVGGNYNYLYRLFRKSNL
KPFERDISTEIYQAGSTPCNGVEGFNCYFPLQSYGFQPTNGVGYQPYRVVVLSFELLHAPATVCGPKKSTNLVKNKCVN
FNFNGLTGTGVLTESNKKFLPFQQFGRDIADTTDAVRDPQTLEILDITPCSFGGVSVITPGTNTSNQVAVLYQDVNCTEV
PVAIHADQLTPTWRVYSTGSNVFQTRAGCLIGAEHVNNSYECDIPIGAGICASYQTQTNSPSGAGSVASQSIIAYTMSLG
AENSVAYSNNSIAIPTNFTISVTTEILPVSMTKTSVDCTMYICGDSTECSNLLLQYGSFCTQLNRALTGIAVEQDKNTQEV
FAQVKQIYKTPPIKDFGGFNFSQILPDPSKPSKRSFIEDLLFNKVTLADAGFIKQYGDCLGDIAARDLICAQKFNGLTVLPP
LLTDEMIAQYTSALLAGTITSGWTFGAGAALQIPFAMQMAYRFNGIGVTQNVLYENQKLIANQFNSAIGKIQDSLSSTASA
LGKLQDVVNQNAQALNTLVKQLSSNFGAISSVLNDILSRLDPPEAEVQIDRLITGRLQSLQTYVTQQLIRAAEIRASANLAA
TKMSECVLGQSKRVDFCGKGYHLMSFPQSAPHGVVFLHVTYVPAQEKNFTTAPAICHDGKAHFPREGVFVSNGTHWF
VTQRNFYEPQIITTDNTFVSGNCDVVIGIVNNTVYDPLQPELDSFKEELDKYFKNHTSPDVDLGDISGINASVVNIQKEIDR
LNEVAKNLNESLIDLQELGKYEQYIKGSGRENLYFQGGGGSGYIPEAPRDGQAYVRKDGEWVLLSTFLGHHHHHHHH

>hACE2(R518G)-Fc
MARAWIFFLLCLAGRALASTIEEQAKTFLDKFNHEAEDLFYQSSLASWNYNTNITEENVQNMNNAGDKWSAFLKEQSTL
AQMYPLQEIQNLTVKLQLQALQQNGSSVLSEDKSKRLNTILNTMSTIYSTGKVCNPDNPQECLLLEPGLNEIMANSLDYN
ERLWAWESWRSEVGKQLRPLYEEYVVLKNEMARANHYEDYGDYWRGDYEVNGVDGYDYSRGQLIEDVEHTFEEIKP
LYEHLHAYVRAKLMNAYPSYISPIGCLPAHLLGDMWGRFWTNLYSLTVPFGQKPNIDVTDAMVDQAWDAQRIFKEAEKF
FVSVGLPNMTQGFWENSMLTDPGNVQKAVCHPTAWDLGKGDFRILMCTKVTMDDFLTAHHEMGHIQYDMAYAAQPF
LLRNGANEGFHEAVGEIMSLSAATPKHLKSIGLLSPDFQEDNETEINFLLKQALTIVGTLPFTYMLEKWRWMVFKGEIPK
DQWMKKWWEMKREIVGVVEPVPHDETYCDPASLFHVSNDYSFIRYYTGTLYQFQFQEALCQAAKHEGPLHKCDISNS
TEAGQKLFNMLRLGKSEPWTLALENVVGAKNMNVRPLLNYFEPLFTWLKDQNKNSFVGWSTDWSPYADPLVPRGSG
GGGDPEPKSCDKTHTCPPCPAPELLGGPSVFLFPPKPKDTLMISRTPEVTCVVVDVSHEDPEVKFNWYVDGVEVHNAK
TKPREEQYNSTYRVVSVLTVLHQDWLNGKEYKCKVSNKALPAPIEKTISKAKGQPREPQVYTLPPSRDELTKNQVSLTC
LVKGFYPSDIAVEWESNGQPENNYKTTPPVLDSDGSFFLYSKLTVDKSRWQQGNVFSCSVMHEALHNHYTQKSLSLSP
GK

>mACE2-Fc
MPMGSLQPLATLYLLGMLVASVLAQSTIEEQAKTFLDKFNHEAEDLFYQSSLASWNYNTNITEENVQNMNNAGEKWSA
FLKEQSTLAQMYPLQEIQNLTVKLQLQALQQNGSSVLSEDKSKRLNTILNTMSTIYSTGKVCNPNNPQECLLLDPGLNEI
MEKSLDYNERLWAWEGWRSEVGKQLRPLYEEYVVLKNEMARANHYKDYGDYWRGNYEVNGVDGYDYNRDQLIEDV
ERTFEEIKPLYEHLHAYVRAKLMNAYPSYISPTGCLPAHLLGDMWGRFWTNLYSLTVPFGQKPNIDVTDAMVNQAWNA
QRIFKEAEKFFVSVGLPNMTQGFWENSMLTDPGNVQKVVCHPTAWDLGKGDFRIIMCTKVTMDDFLTAHHEMGHIQYD
MAYAAQPFLLRNGANEGFHEAVGEIMSLSAATPKHLKSIGLLSPDFQEDNETEINFLLKQALTIVGTLPFTYMLEKWRWM
VFKGEIPKDQWMKKWWEMKREIVGVVEPVPHDETYCDPASLFHVSNDYSFIRYYTRTLYQFQFQEALCQAAKHEGPLH
KCDISNSTEAGQKLLNMLKLGKSEPWTLALENVVGAKNMNVRPLLNYFEPLFTWLKDQNKNSFVGWSTDWSPYADQSI
KVRISLKSALGDKAYEWNDNEMYLFRSSVAYAMRTYFLEIKHQTILFGEEDVRVADLKPRISFNFYVTAPKNVSDIIPRTE
VEEAIRISRSRINDAFRLNDNSLEFLGIQTTLAPPYQSPVTDPLVPRGSGGGGDPEPKSCDKTHTCPPCPAPELLGGPSV
FLFPPKPKDTLMISRTPEVTCVVVDVSHEDPEVKFNWYVDGVEVHNAKTKPREEQYNSTYRVVSVLTVLHQDWLNGKE
YKCKVSNKALPAPIEKTISKAKGQPREPQVYTLPPSRDELTKNQVSLTCLVKGFYPSDIAVEWESNGQPENNYKTTPPVL
DSDGSFFLYSKLTVDKSRWQQGNVFSCSVMHEALHNHYTQKSLSLSPGK
