# Supplementary material for: Elicitation of Potent Neutralizing Antibody Responses by Designed Protein Nanoparticle Vaccines for SARS-CoV-2
Source: Cell. 2020 Nov 25;183(5):1367–1382.e17. doi: 10.1016/j.cell.2020.10.043 (PMC7604136; doi:10.1016/j.cell.2020.10.043)
Supplement: Data S2. Immunogen Stability Data, Related to Figure 3 [file mmc2.pdf]

Elicitation of potent neutralizing antibody responses by designed protein nanoparticle vaccines for SARS-CoV-2

Walls AC & Fiala B, et al.

Supplemental Stability Data

SDS-PAGE for Monomeric RBD

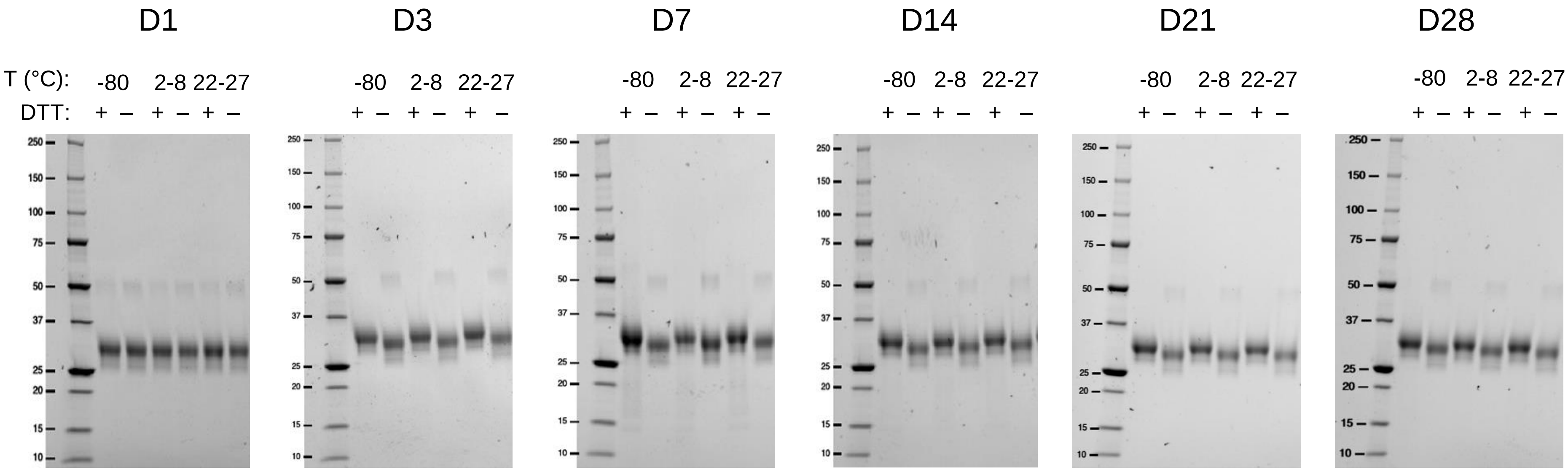

The integrity of samples incubated at three different temperatures for various numbers of days (D) was analyzed by SDS-PAGE. Molecular weights of the standard are noted in kDa. Each sample was analyzed +/- DTT.

mACE2-Fc Binding for Monomeric RBD

D1

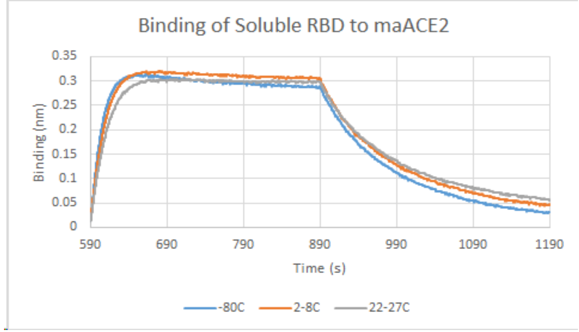

D3

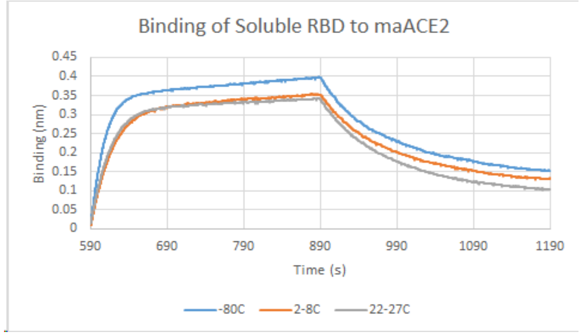

D7

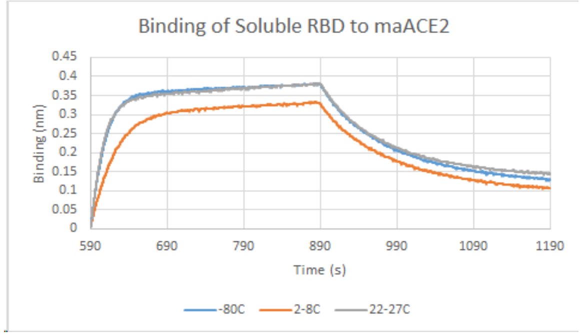

D14

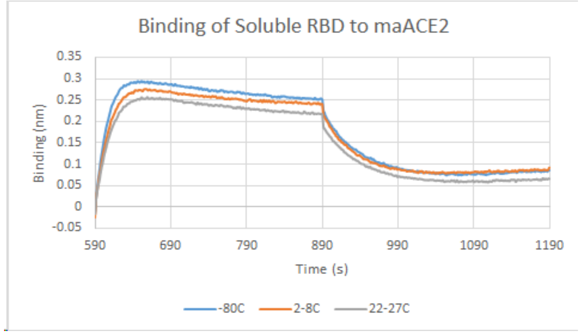

D21

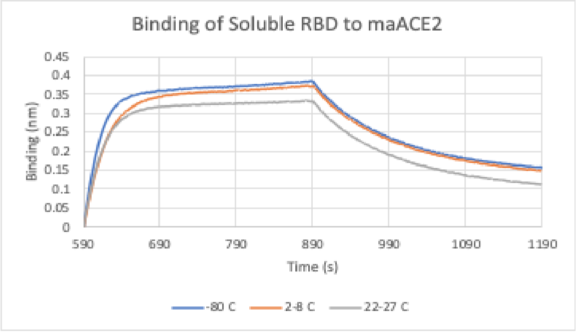

D28

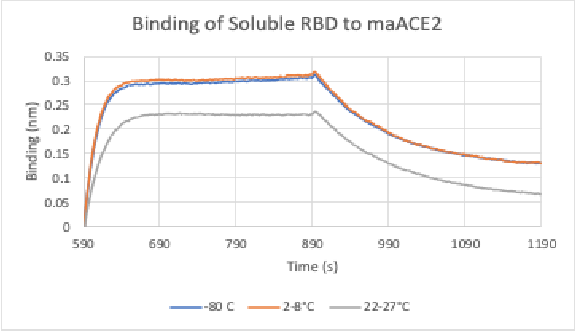

Binding of antigen incubated at three different temperatures for various numbers of days (D) to mACE2-Fc was analyzed by Biolayer Interferometry (BLI). Protein A biosensors loaded with mACE2-Fc were incubated with antigen (association, x = 590–889 s) and then buffer (dissociation, x = 890–1190 s).

CR3022 IgG Binding for Monomeric RBD

D1

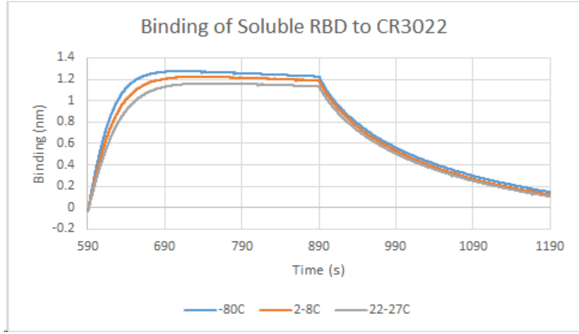

D3

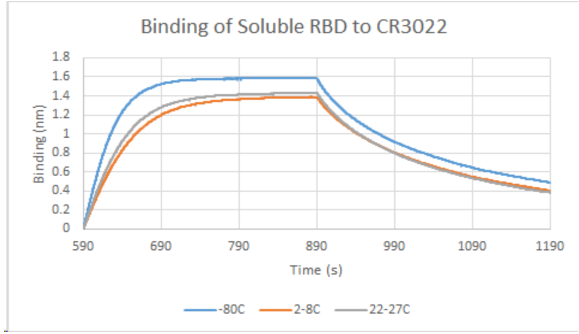

D7

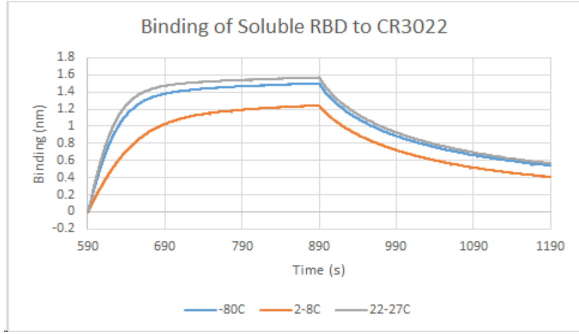

D14

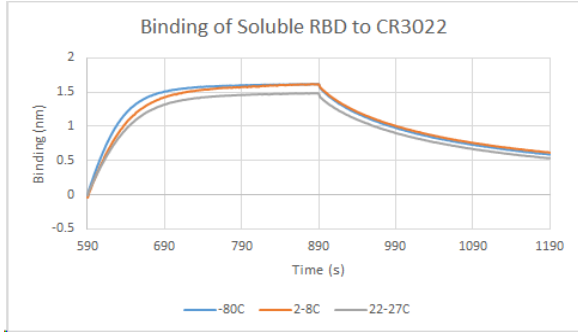

D21

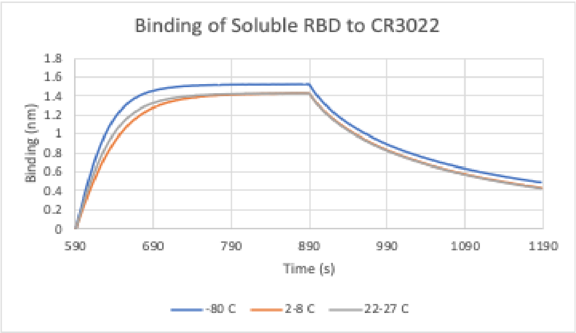

D28

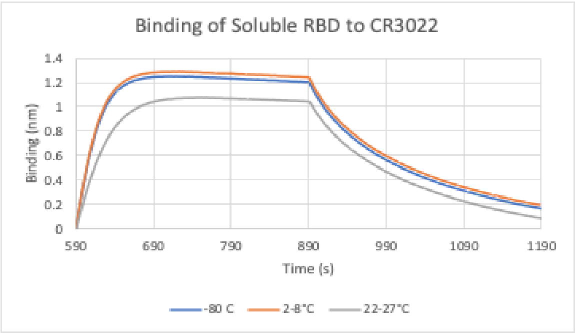

Binding of antigen incubated at three different temperatures for various numbers of days (D) to CR3022 was analyzed by Biolayer Interferometry (BLI). Protein A biosensors loaded with CR3022 were incubated with antigen (association, x = 590–889 s) and then buffer (dissociation, x = 890–1190 s).

Absorbance at 320/280 nm for Monomeric RBD

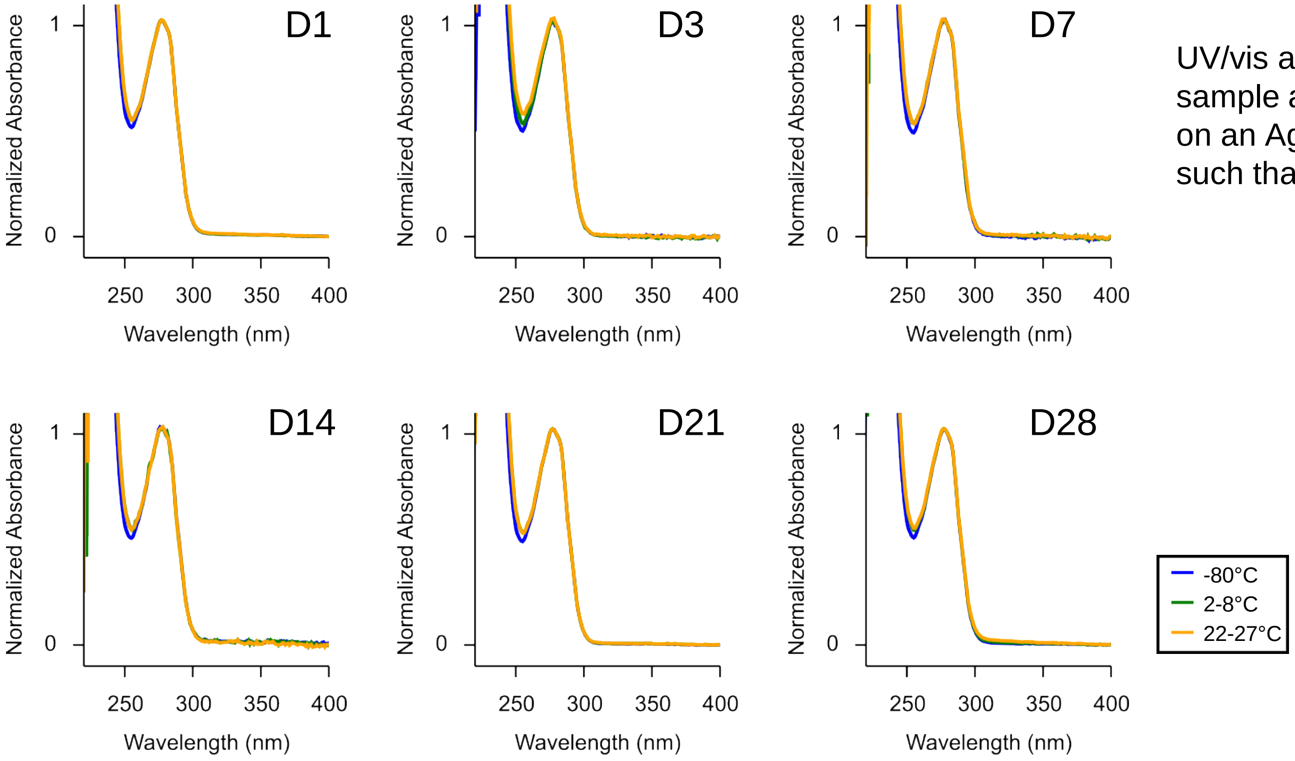

UV/vis absorbance spectra for each sample at each time point were obtained on an Agilent Cary 8454 and normalized such that $A_{280} = 1$ and $A_{400} = 0$.

SDS-PAGE for S-2P trimer

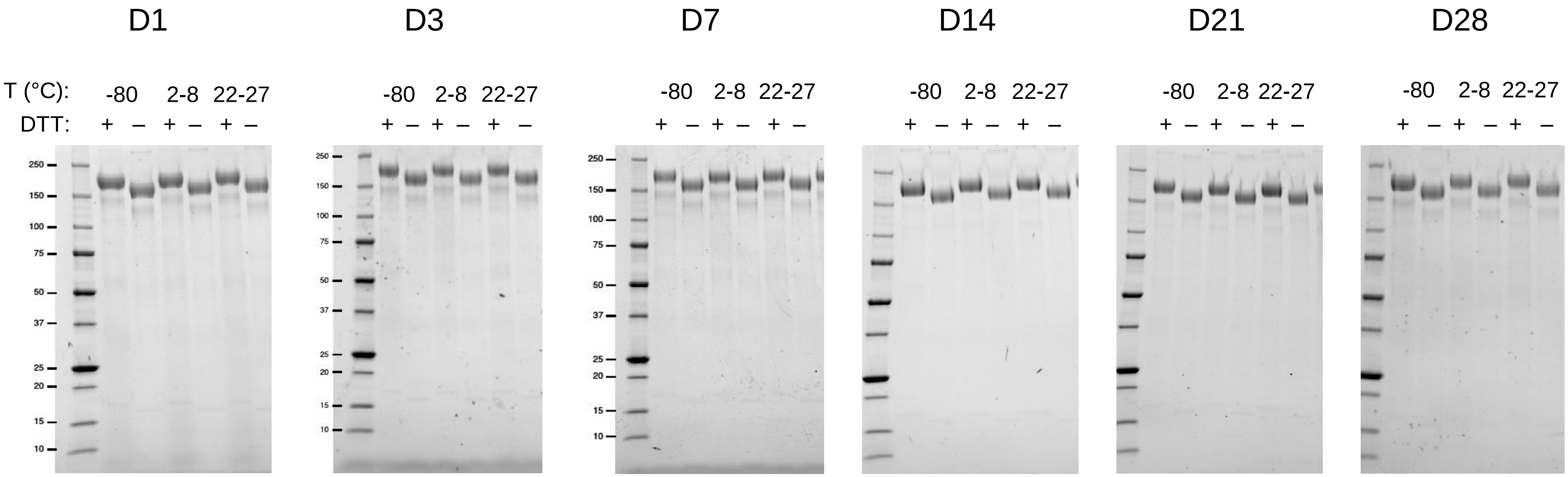

The integrity of samples incubated at three different temperatures for various numbers of days (D) was analyzed by SDS-PAGE. Molecular weights of the standard are noted in kDa. Each sample was analyzed +/- DTT.

nsEM for S-2P Trimer, -80°C

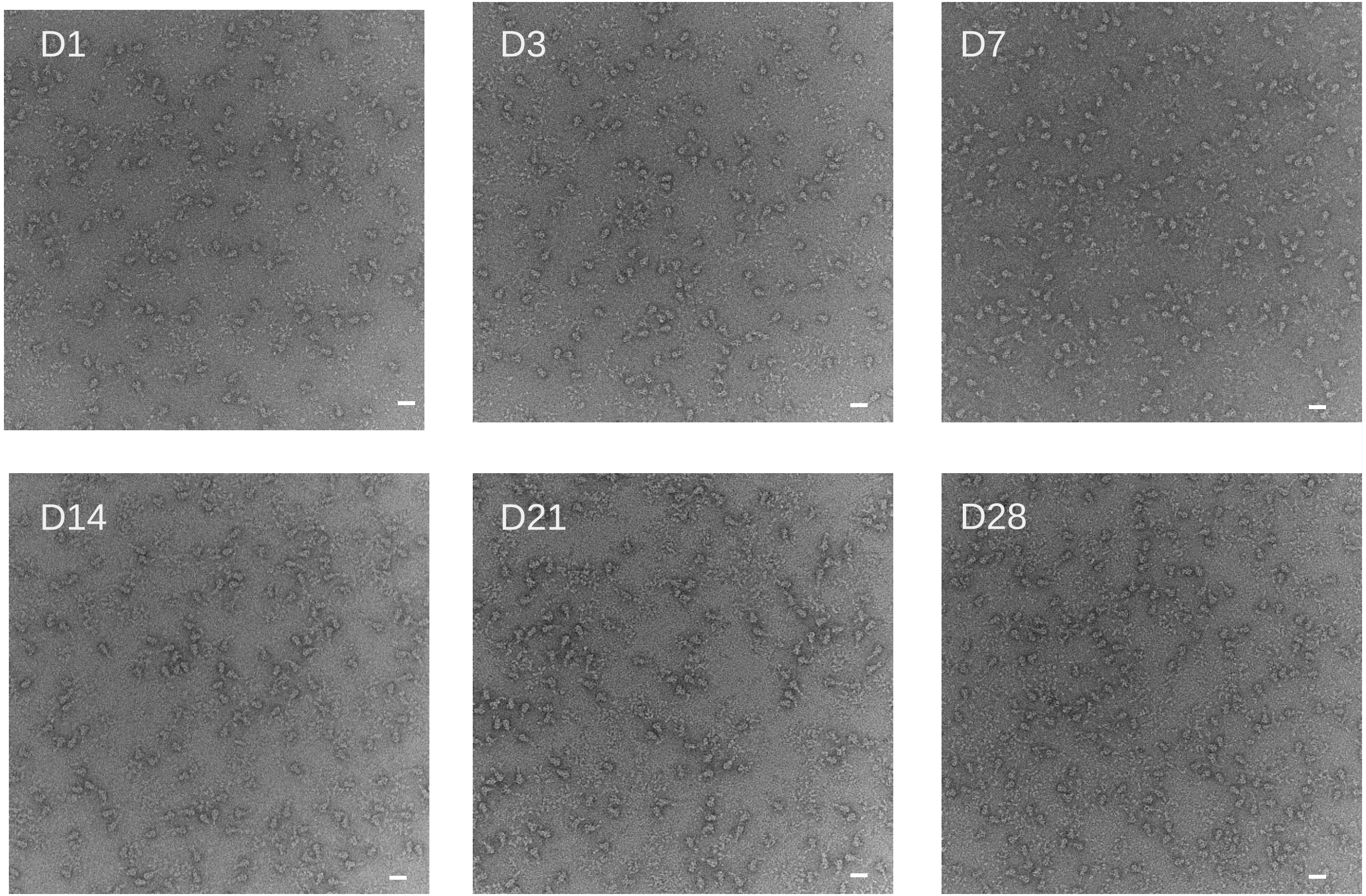

Representative negative stain electron micrographs for each sample at each time point (D, day). Scale bar, 25 nm.

nsEM for S-2P Trimer, 2-8°C

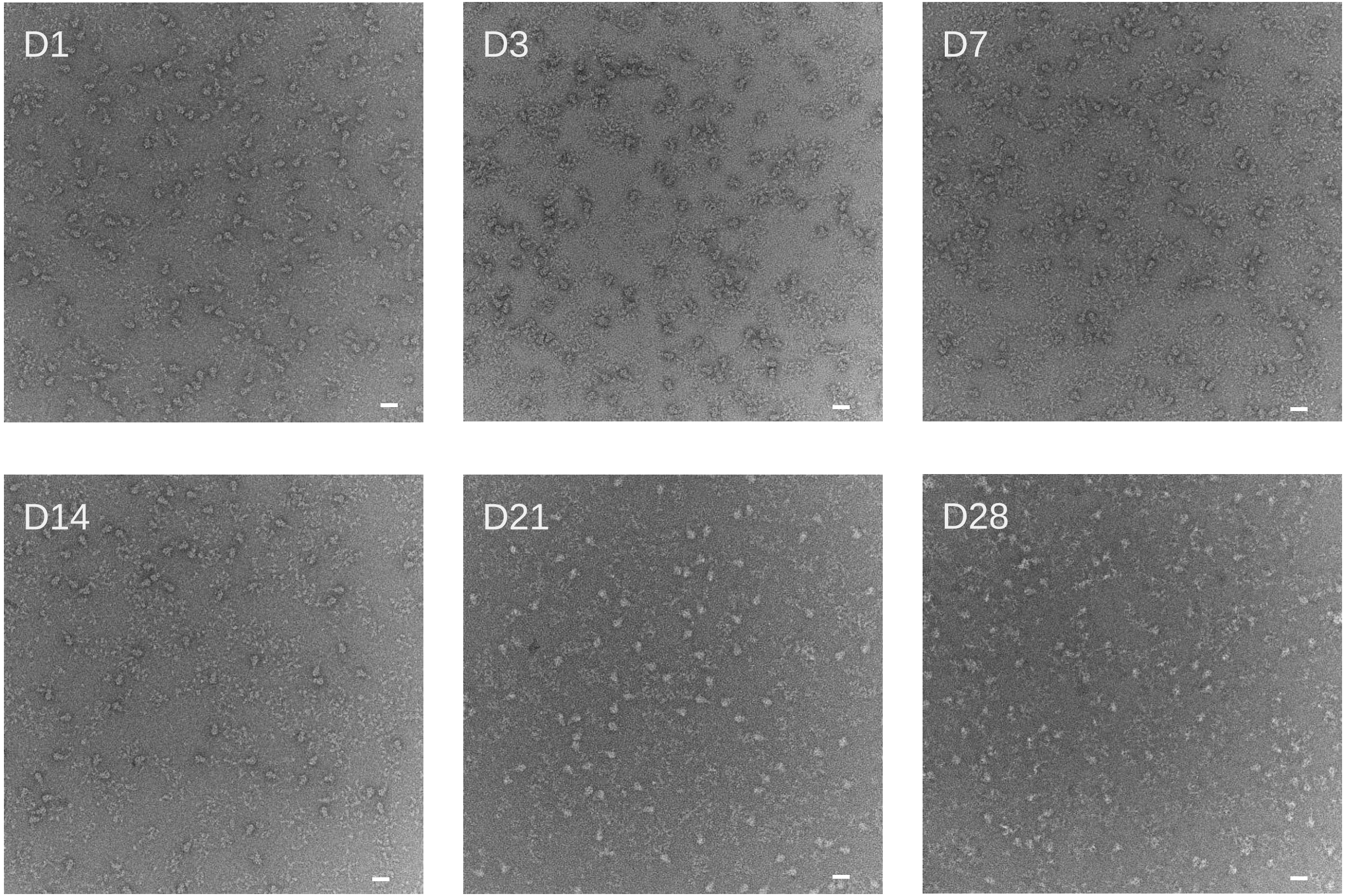

Representative negative stain electron micrographs for each sample at each time point (D, day). Scale bar, 25 nm.

nsEM for S-2P Trimer, 22-27°C

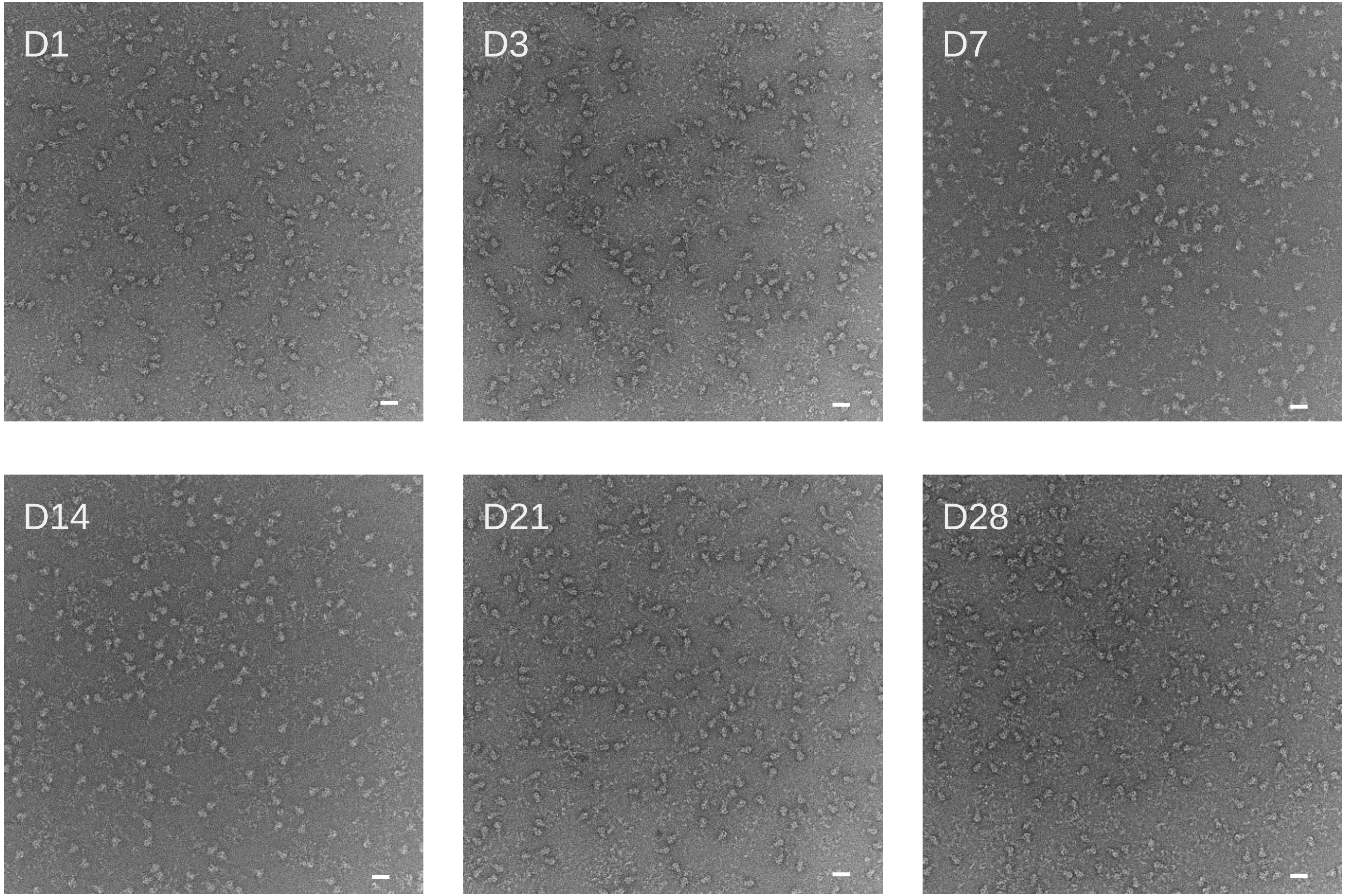

Representative negative stain electron micrographs for each sample at each time point (D, day). Scale bar, 25 nm.

Absorbance at 320/280 for S-2P Trimer

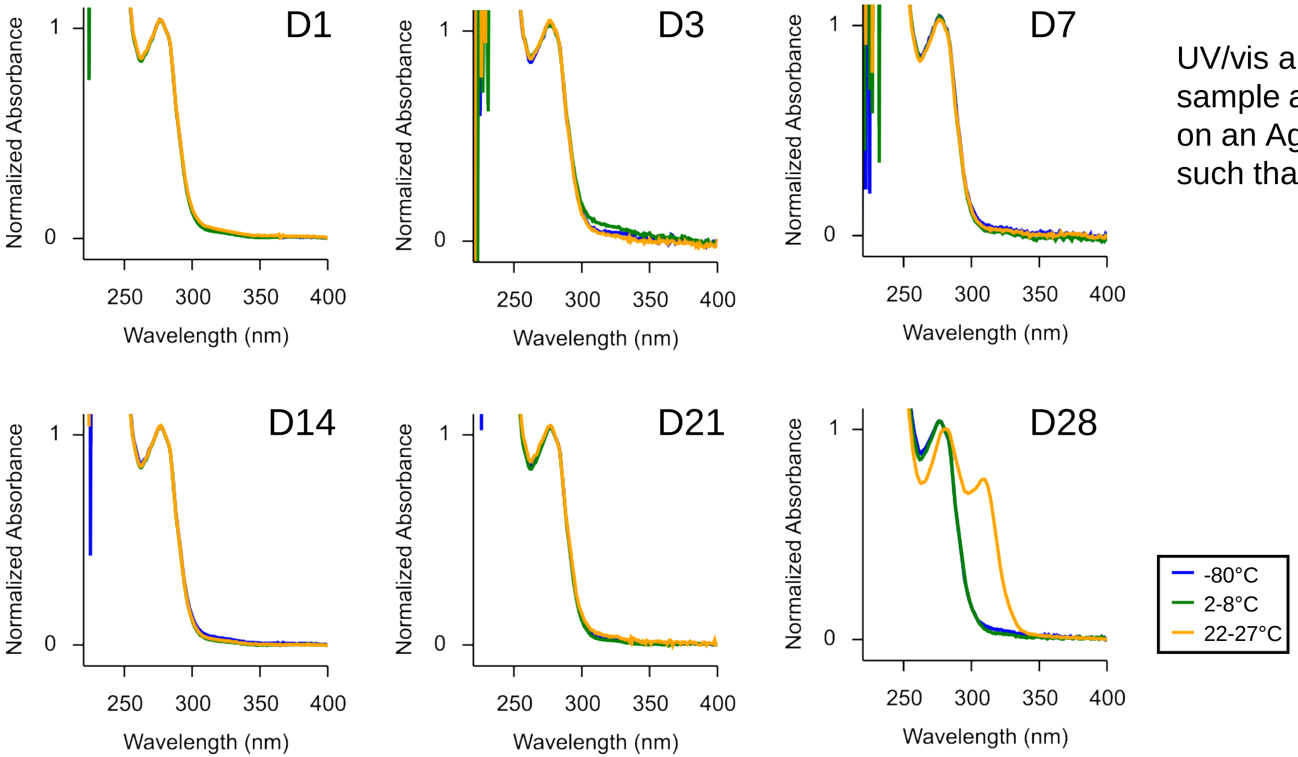

UV/vis absorbance spectra for each sample at each time point were obtained on an Agilent Cary 8454 and normalized such that $A_{280} = 1$ and $A_{400} = 0$.

SDS-PAGE for RBD-8GS-I53-50A component

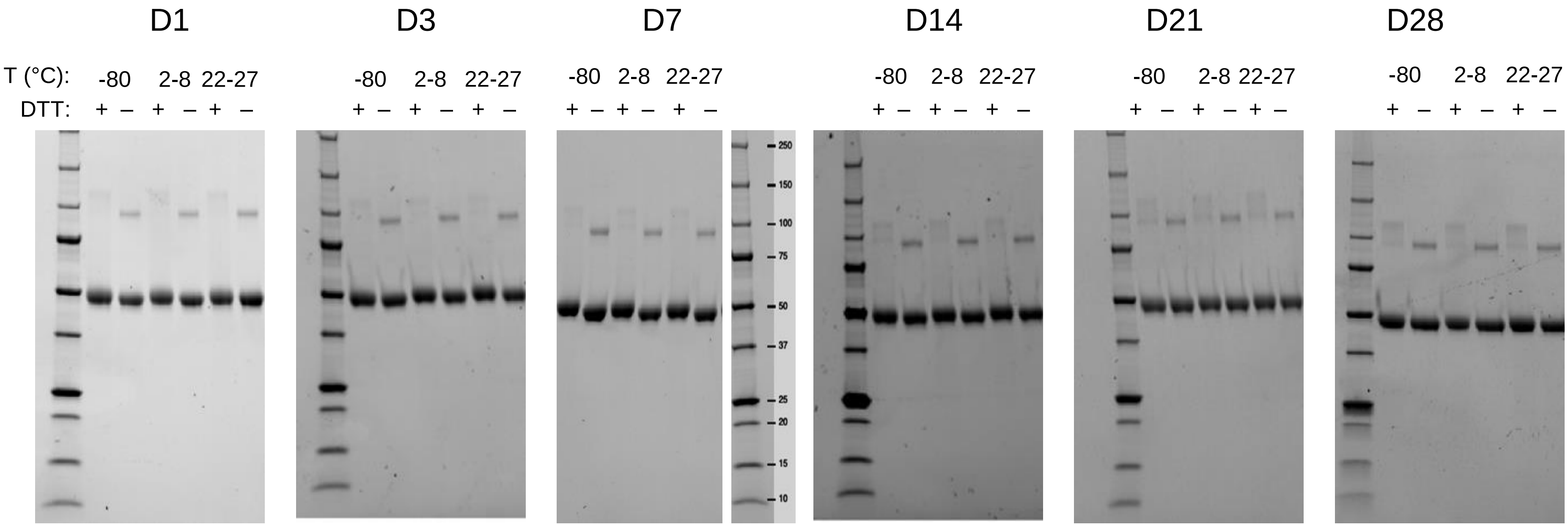

The integrity of samples incubated at three different temperatures for various numbers of days (D) was analyzed by SDS-PAGE. Molecular weights of the standard are noted in kDa. Each sample was analyzed +/- DTT.

mACE2-Fc Binding for RBD-8GS-I53-50A component

D1

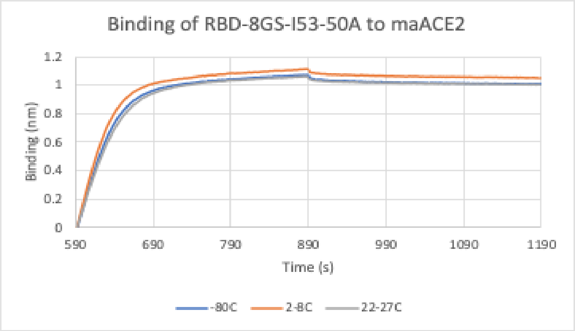

D3

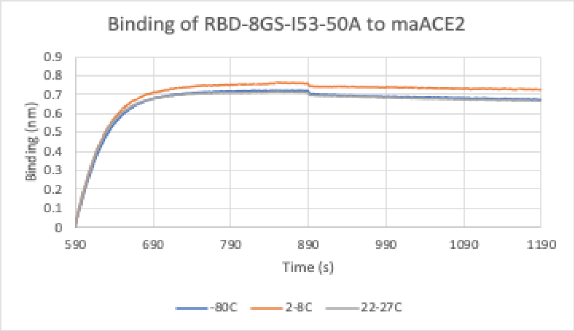

D7

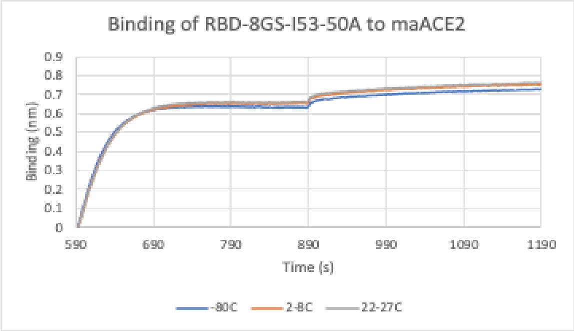

D14

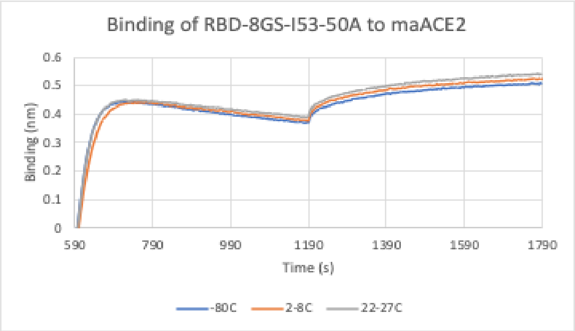

D21

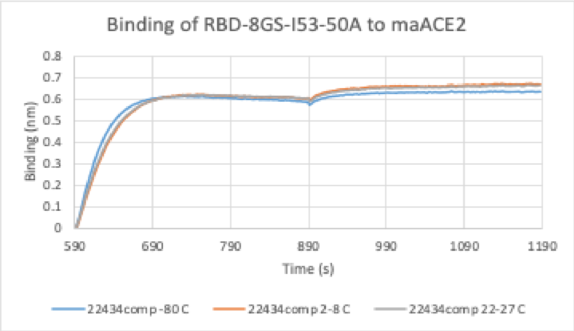

D28

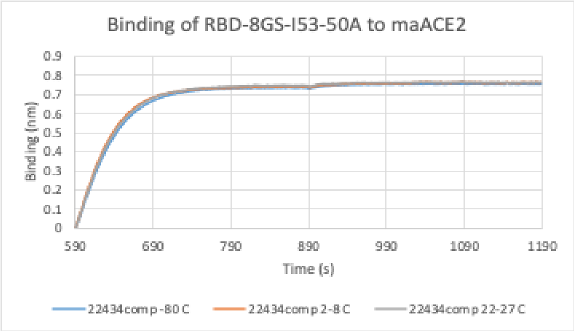

Binding of antigen incubated at three different temperatures for various numbers of days (D) to mACE2-Fc was analyzed by Biolayer Interferometry (BLI). Protein A biosensors loaded with mACE2-Fc were incubated with antigen (association, x = 590–889 s) and then buffer (dissociation, x = 890–1190 s).

CR3022 IgG for RBD-8GS-I53-50A component

D1

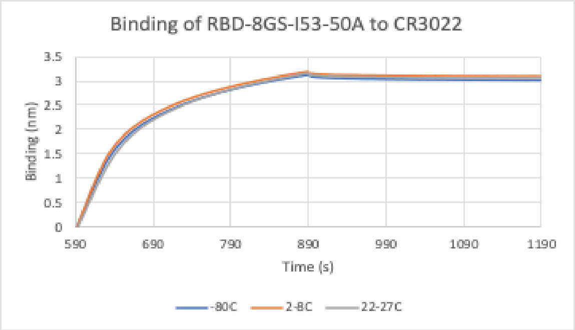

D3

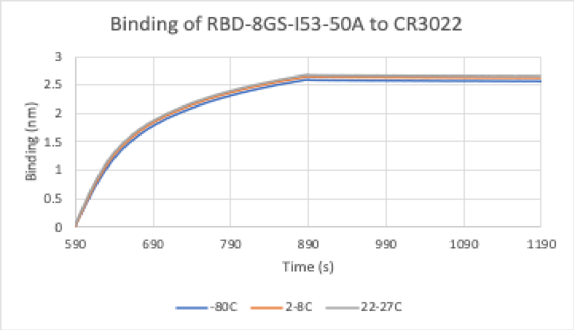

D7

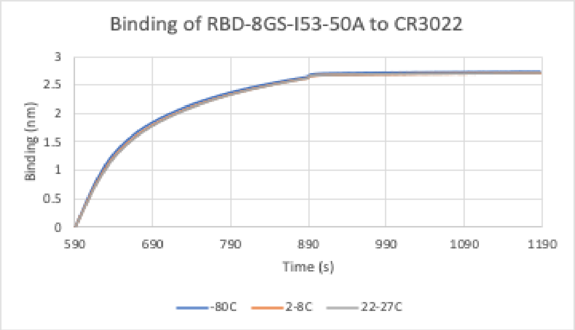

D14

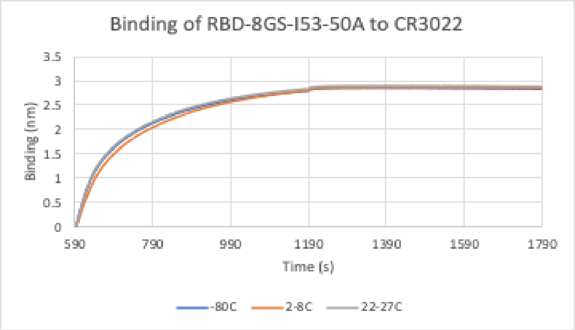

D21

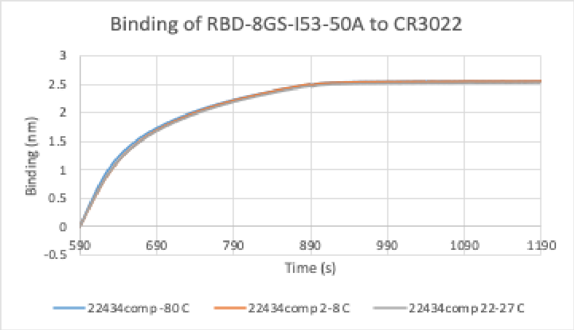

D28

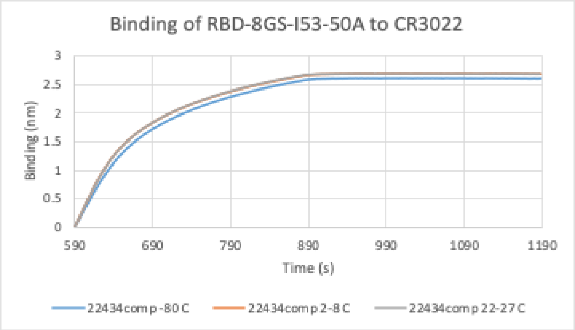

Binding of antigen incubated at three different temperatures for various numbers of days (D) to CR3022 was analyzed by Biolayer Interferometry (BLI). Protein A biosensors loaded with CR3022 were incubated with antigen (association, x = 590–889 s) and then buffer (dissociation, x = 890–1190 s).

Absorbance at 320/280 for RBD-8GS-I53-50A component

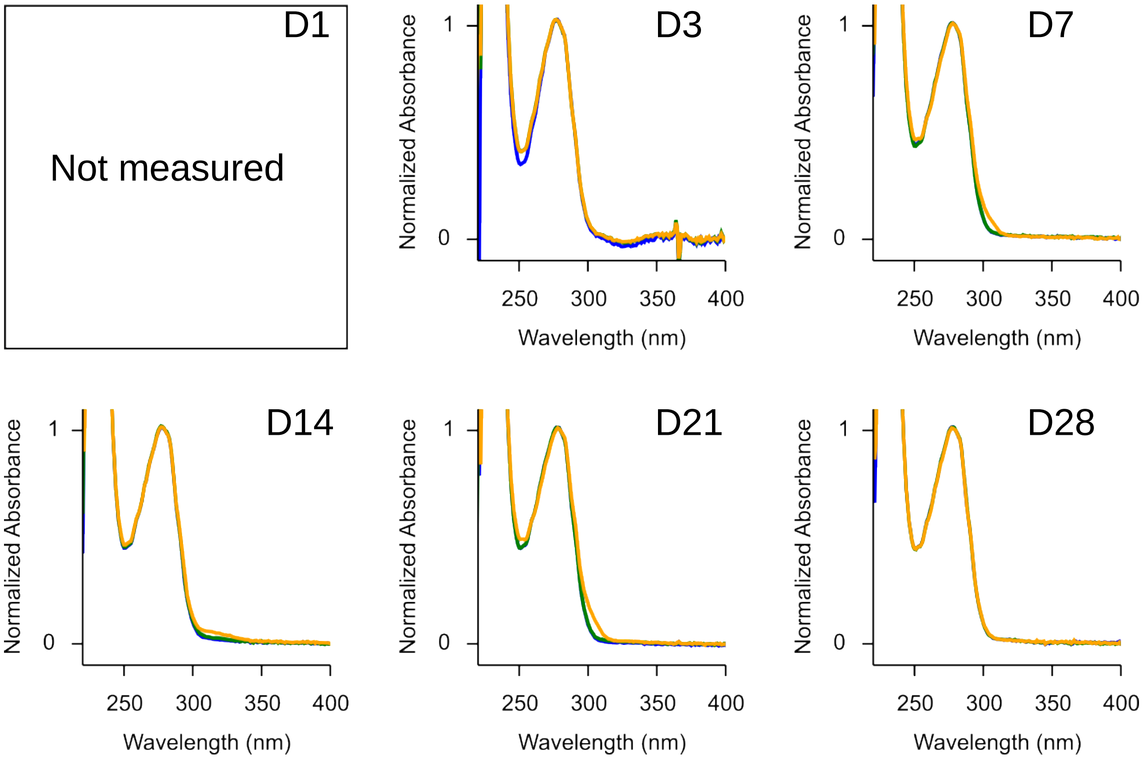

UV/vis absorbance spectra for each sample at each time point were obtained on an Agilent Cary 8454 and normalized such that $A_{280} = 1$ and $A_{400} = 0$.

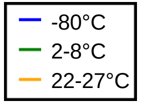

SDS-PAGE for RBD-12GS-I53-50A component

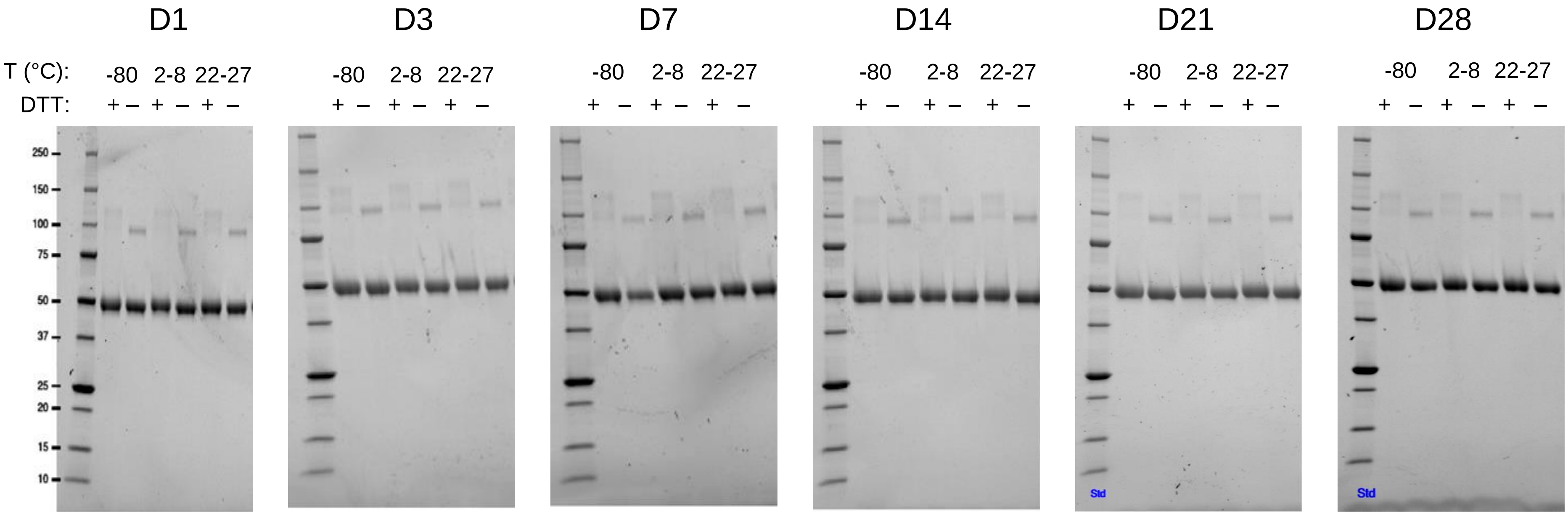

The integrity of samples incubated at three different temperatures for various numbers of days (D) was analyzed by SDS-PAGE. Molecular weights of the standard are noted in kDa. Each sample was analyzed +/- DTT.

mACE2-Fc Binding for RBD-12GS-I53-50A component

D1

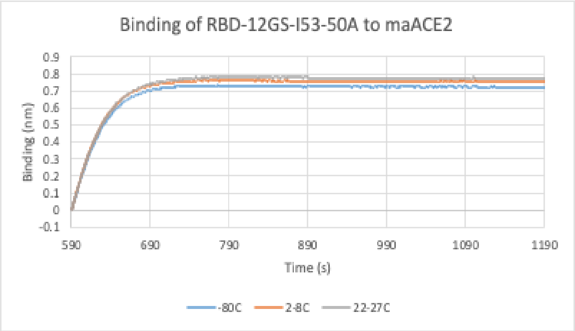

D3

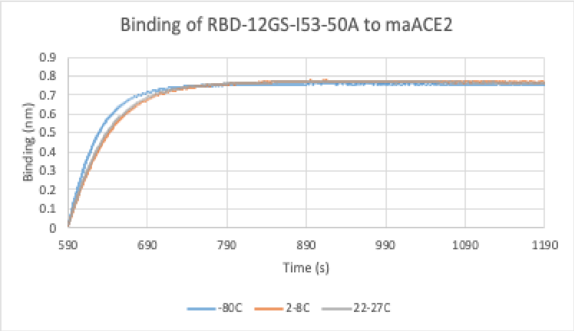

D7

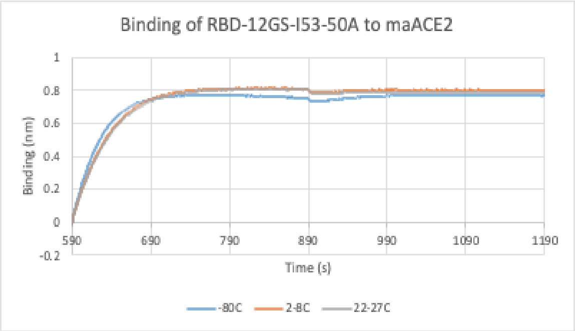

D14

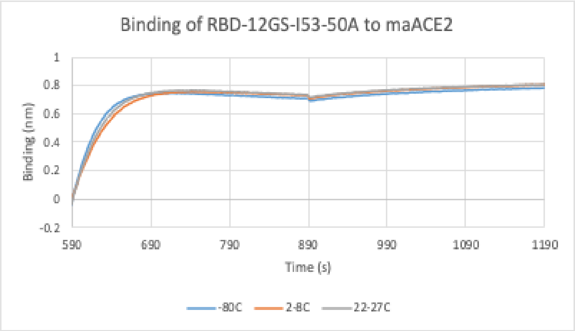

D21

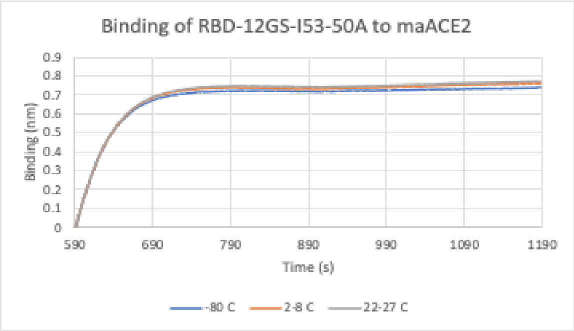

D28

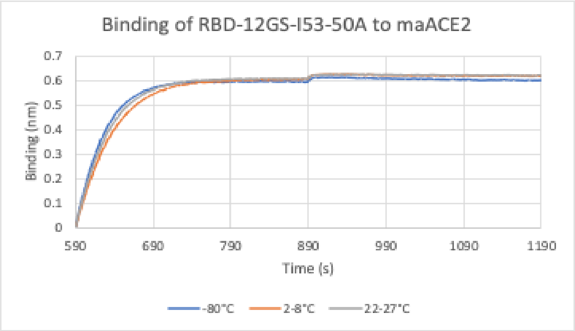

Binding of antigen incubated at three different temperatures for various numbers of days (D) to mACE2-Fc was analyzed by Biolayer Interferometry (BLI). Protein A biosensors loaded with mACE2-Fc were incubated with antigen (association, x = 590–889 s) and then buffer (dissociation, x = 890–1190 s).

CR3022 IgG Binding for RBD-12GS-I53-50A component

D1

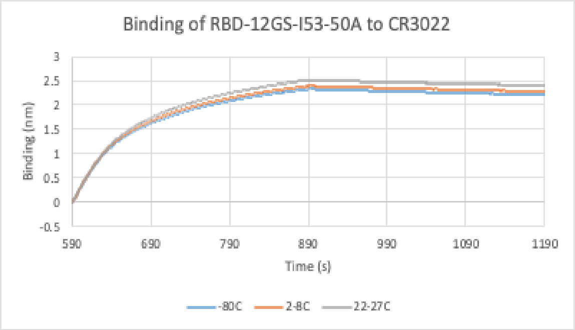

D3

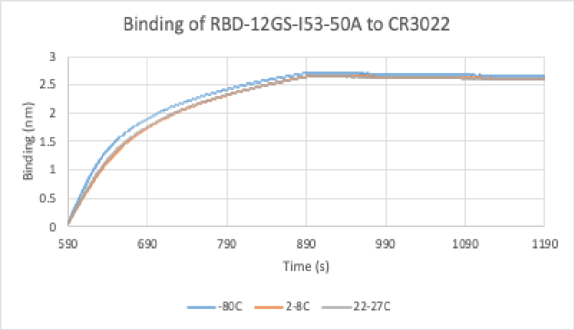

D7

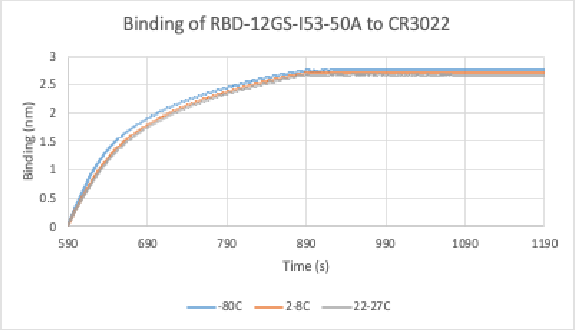

D14

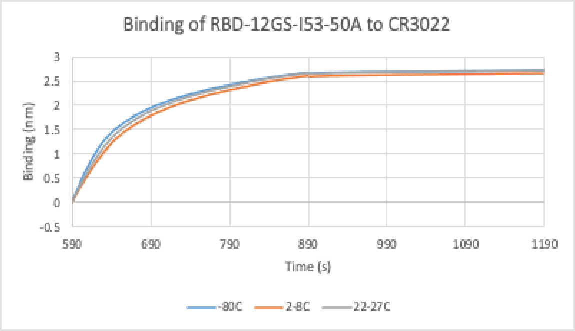

D21

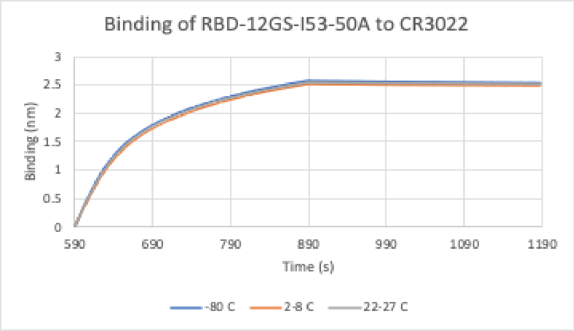

D28

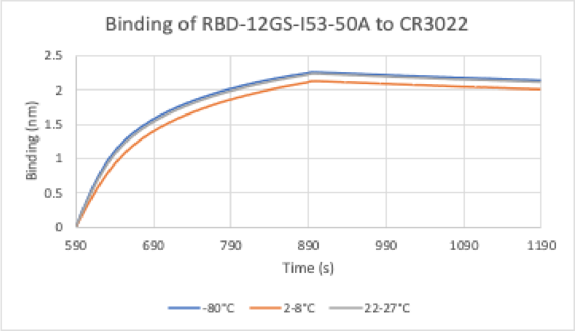

Binding of antigen incubated at three different temperatures for various numbers of days (D) to CR3022 was analyzed by Biolayer Interferometry (BLI). Protein A biosensors loaded with CR3022 were incubated with antigen (association, x = 590–889 s) and then buffer (dissociation, x = 890–1190 s).

Absorbance at 320/280 nm for RBD-12GS-I53-50A component

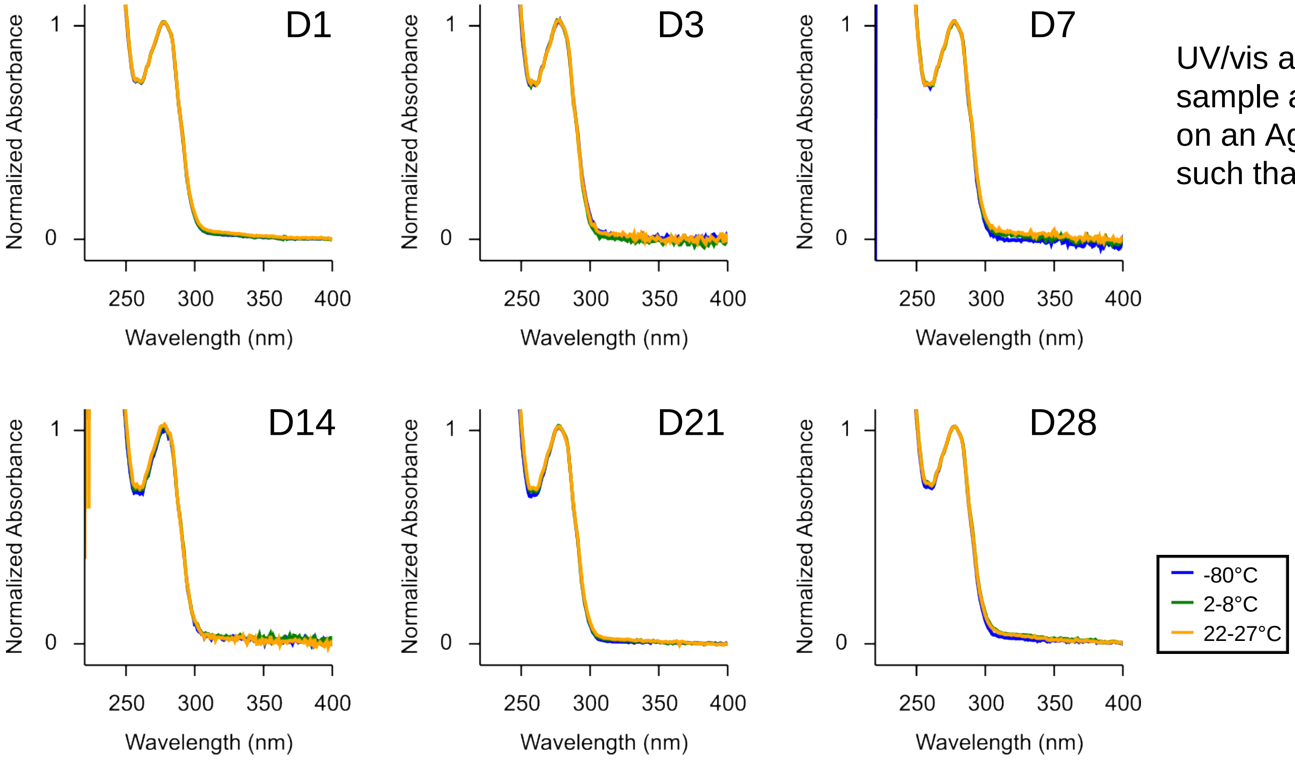

UV/vis absorbance spectra for each sample at each time point were obtained on an Agilent Cary 8454 and normalized such that $A_{280} = 1$ and $A_{400} = 0$.

SDS-PAGE for RBD-16GS-I53-50A component

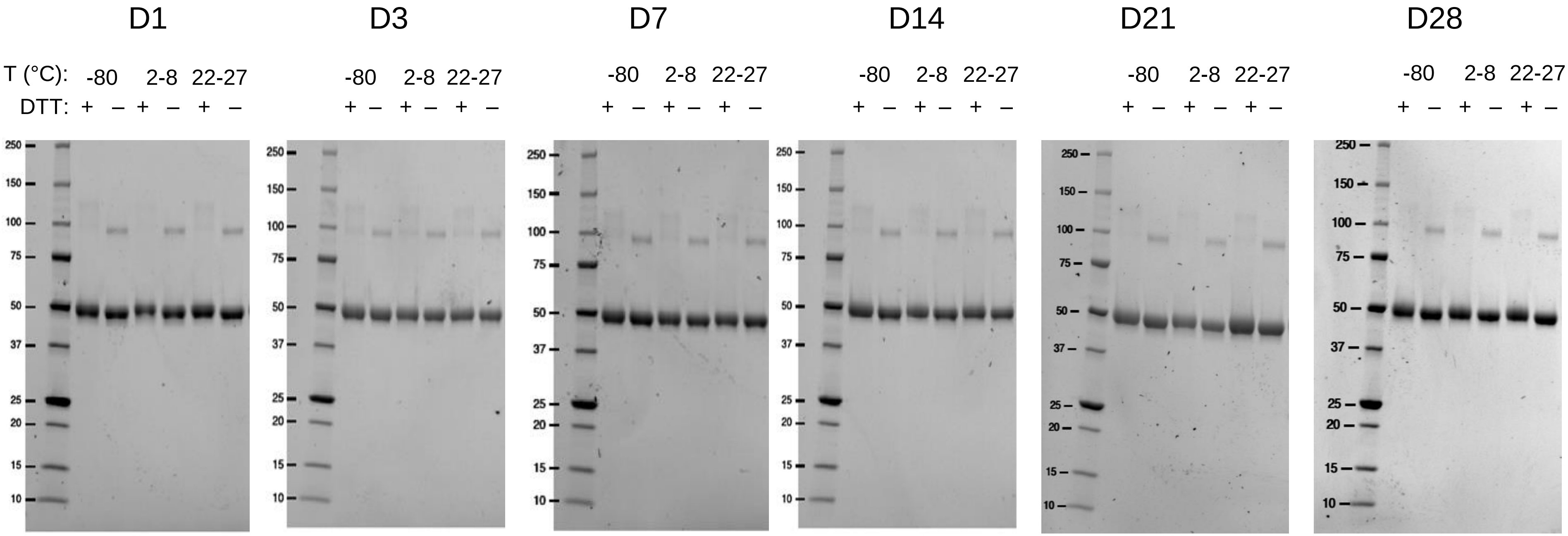

The integrity of samples incubated at three different temperatures for various numbers of days (D) was analyzed by SDS-PAGE. Molecular weights of the standard are noted in kDa. Each sample was analyzed +/- DTT.

mACE2-Fc Binding for RBD-16GS-I53-50A component

D1

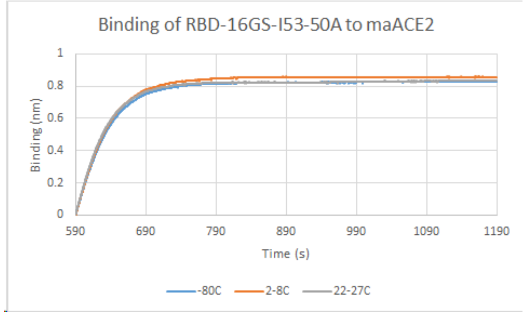

D3

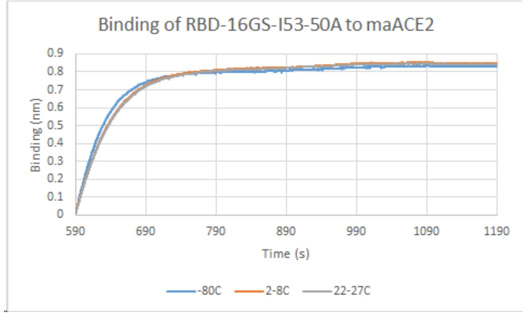

D7

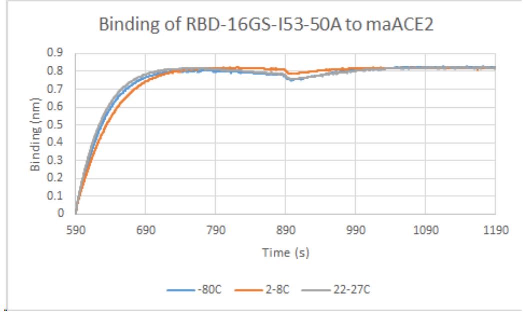

D14

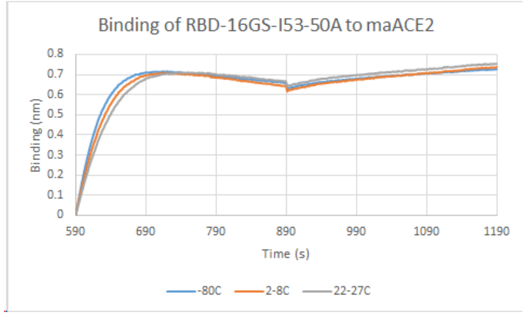

D21

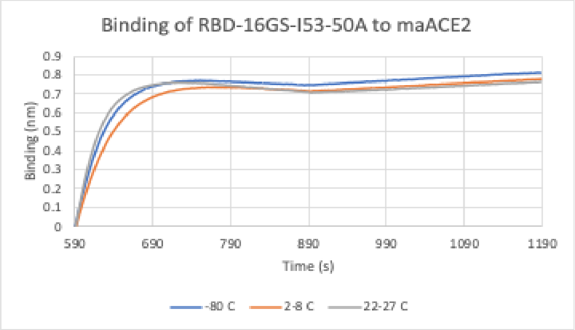

D28

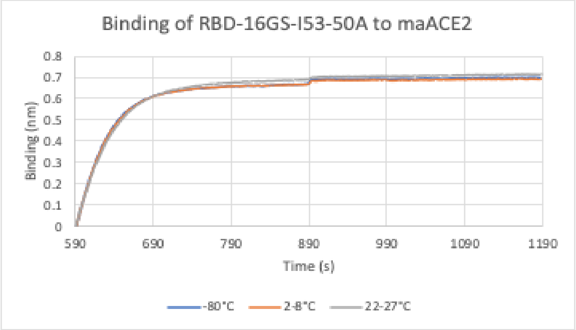

Binding of antigen incubated at three different temperatures for various numbers of days (D) to mACE2-Fc was analyzed by Biolayer Interferometry (BLI). Protein A biosensors loaded with mACE2-Fc were incubated with antigen (association, x = 590–889 s) and then buffer (dissociation, x = 890–1190 s).

CR3022 IgG Binding for RBD-16GS-I53-50A component

D1

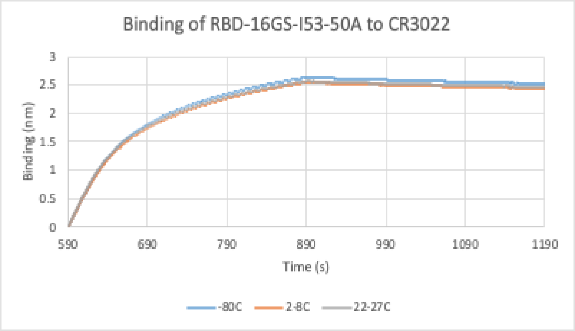

D3

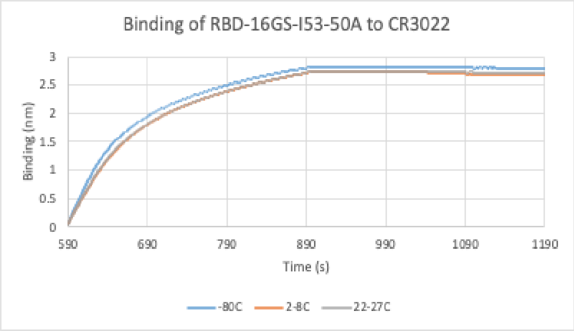

D7

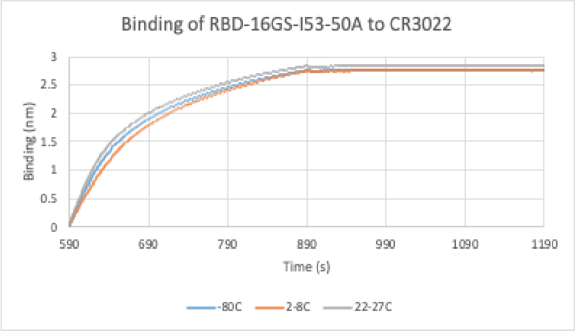

D14

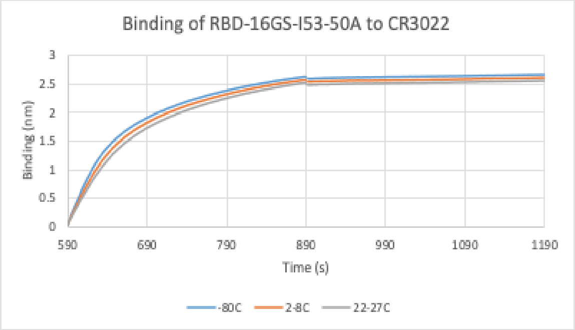

D21

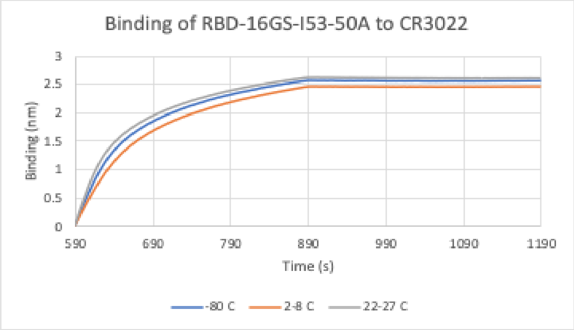

D28

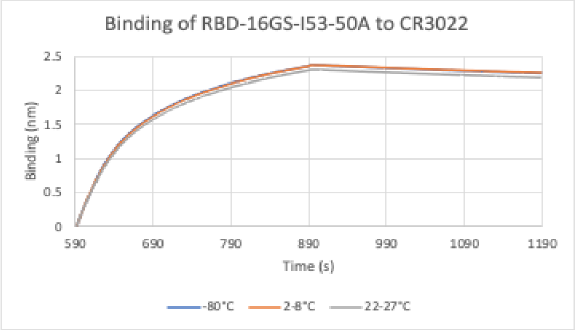

Binding of antigen incubated at three different temperatures for various numbers of days (D) to CR3022 was analyzed by Biolayer Interferometry (BLI). Protein A biosensors loaded with CR3022 were incubated with antigen (association, x = 590–889 s) and then buffer (dissociation, x = 890–1190 s).

Absorbance at 320/280 for RBD-16GS-I53-50A component

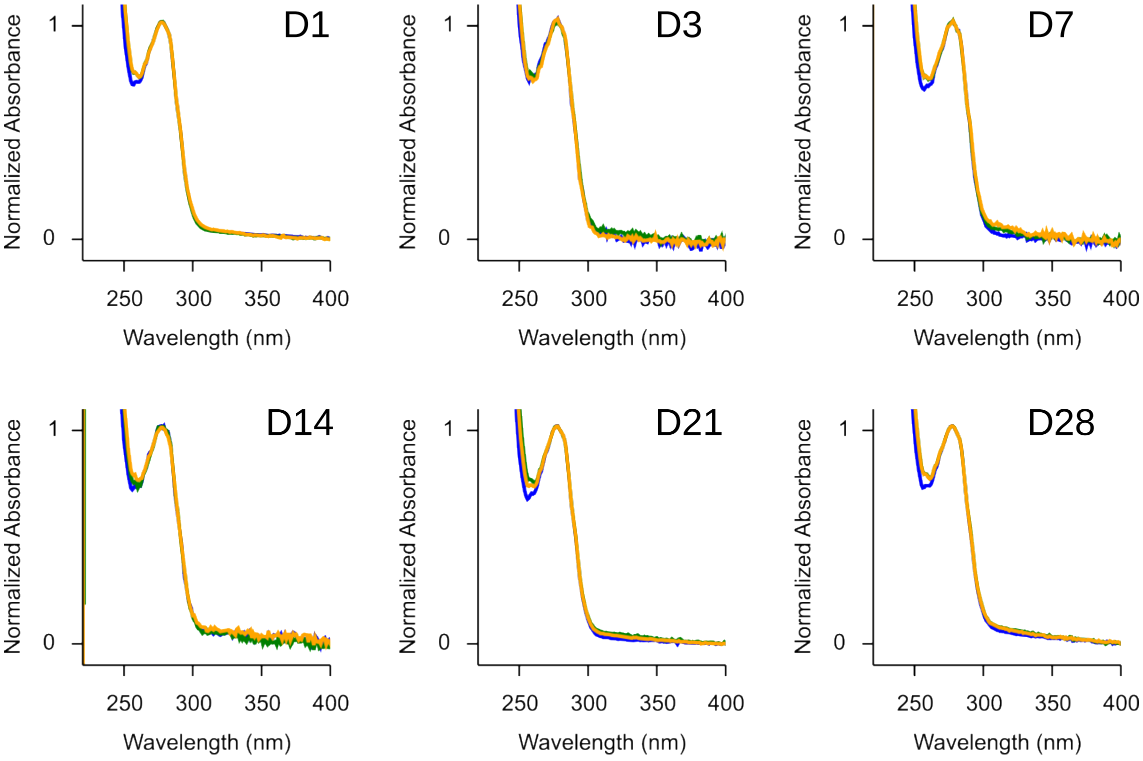

UV/vis absorbance spectra for each sample at each time point were obtained on an Agilent Cary 8454 and normalized such that $A_{280} = 1$ and $A_{400} = 0$.

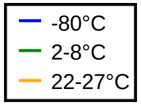

SDS-PAGE for RBD-12GS-I53-50 nanoparticle

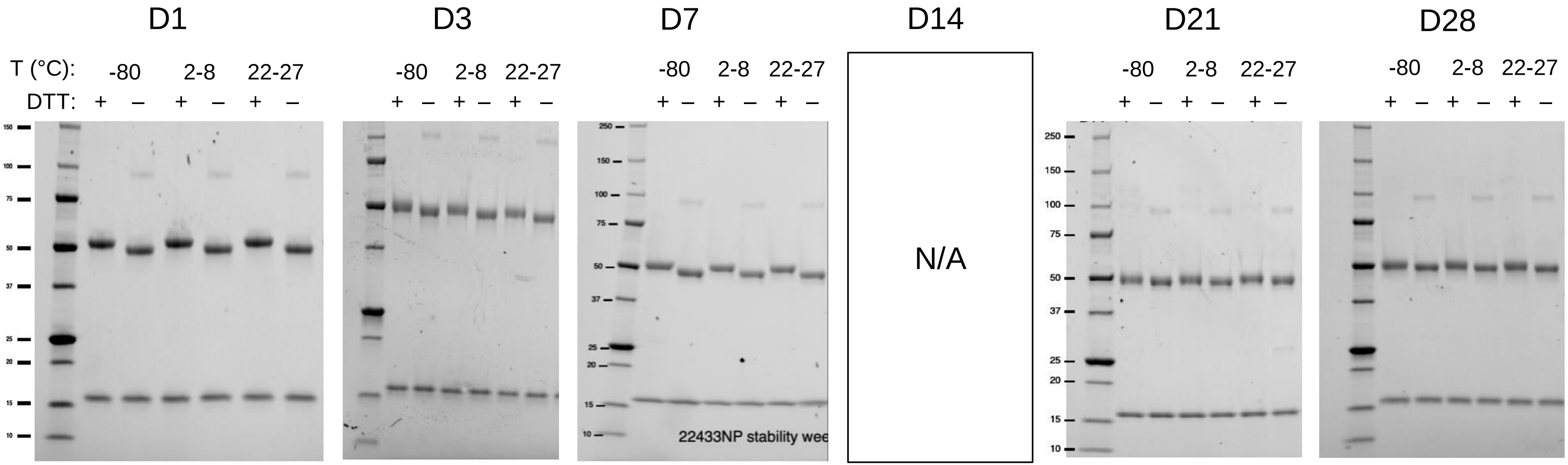

The integrity of samples incubated at three different temperatures for various numbers of days (D) was analyzed by SDS-PAGE. Molecular weights of the standard are noted in kDa. Each sample was analyzed +/- DTT. N/A: Not assessed.

mACE2-Fc Binding for RBD-12GS-I53-50 nanoparticle

D1

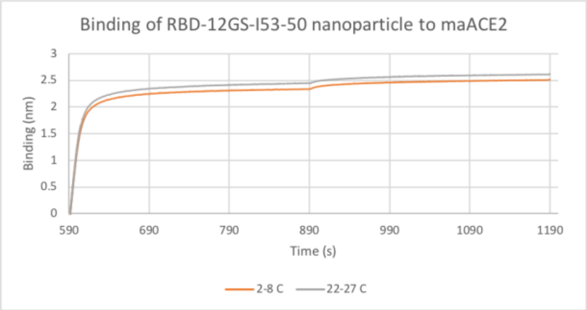

D3

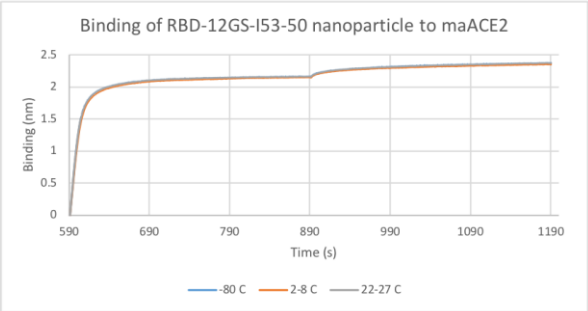

D7

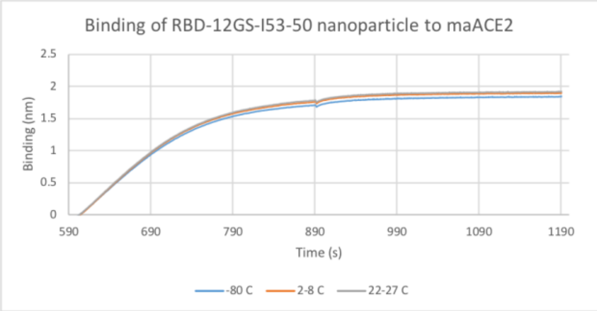

D14

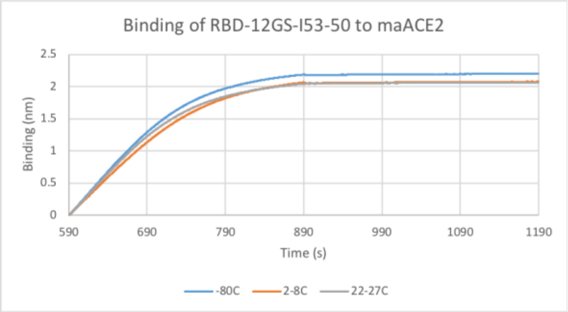

D21

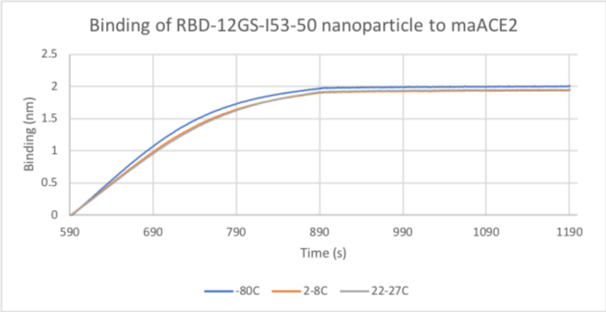

D28

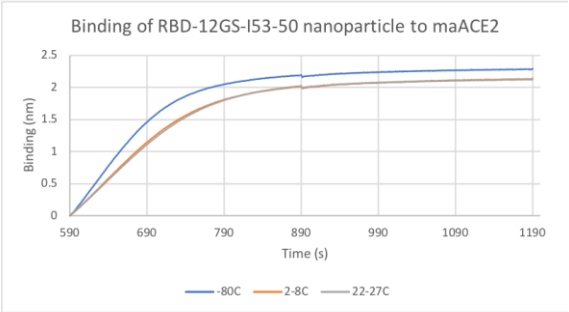

Binding of antigen incubated at three different temperatures for various numbers of days (D) to mACE2-Fc was analyzed by Biolayer Interferometry (BLI). Protein A biosensors loaded with mACE2-Fc were incubated with antigen (association, x = 590–889 s) and then buffer (dissociation, x = 890–1190 s).

CR3022 IgG Binding for RBD-12GS-I53-50 nanoparticle

D1

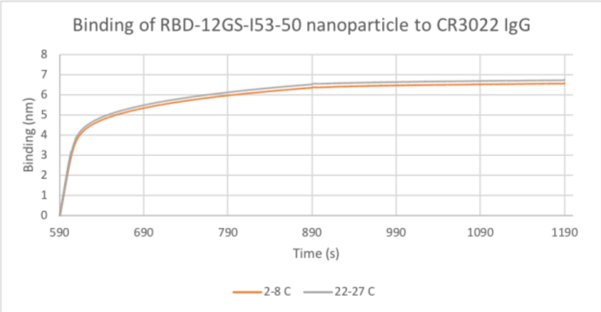

D3

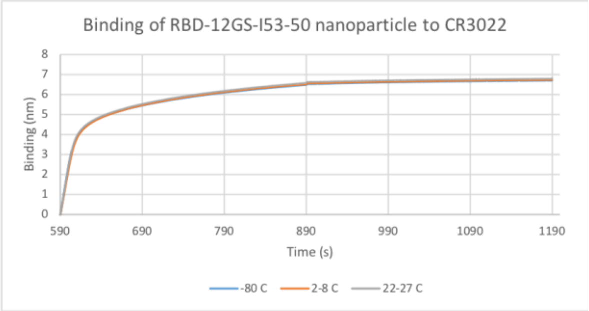

D7

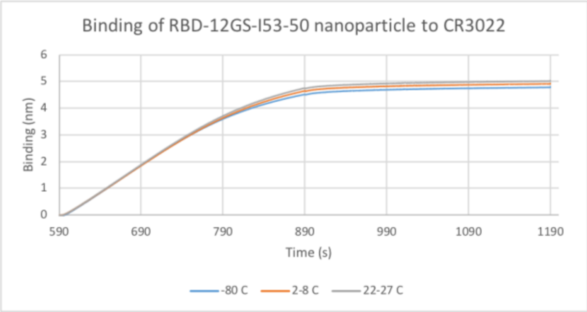

D14

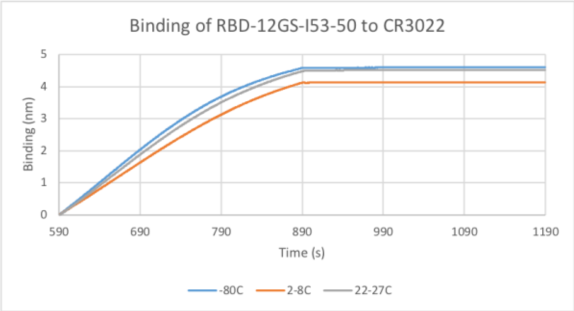

D21

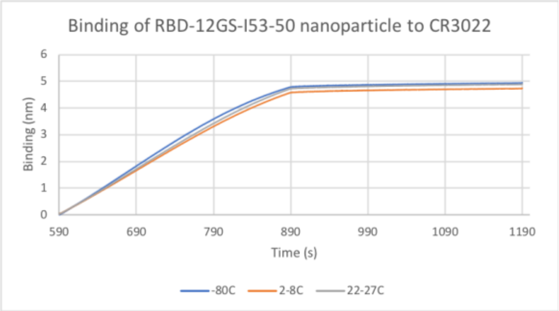

D28

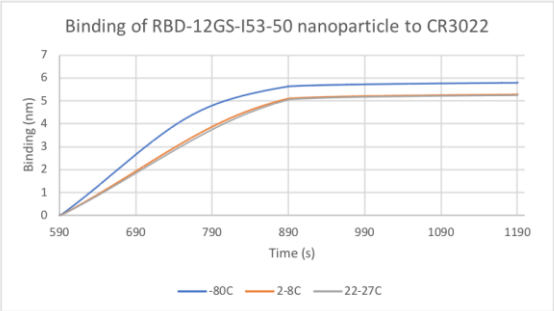

Binding of antigen incubated at three different temperatures for various numbers of days (D) to CR3022 was analyzed by Biolayer Interferometry (BLI). Protein A biosensors loaded with CR3022 were incubated with antigen (association, x = 590–889 s) and then buffer (dissociation, x = 890–1190 s).

nsEM for RBD-12GS-I53-50 nanoparticle

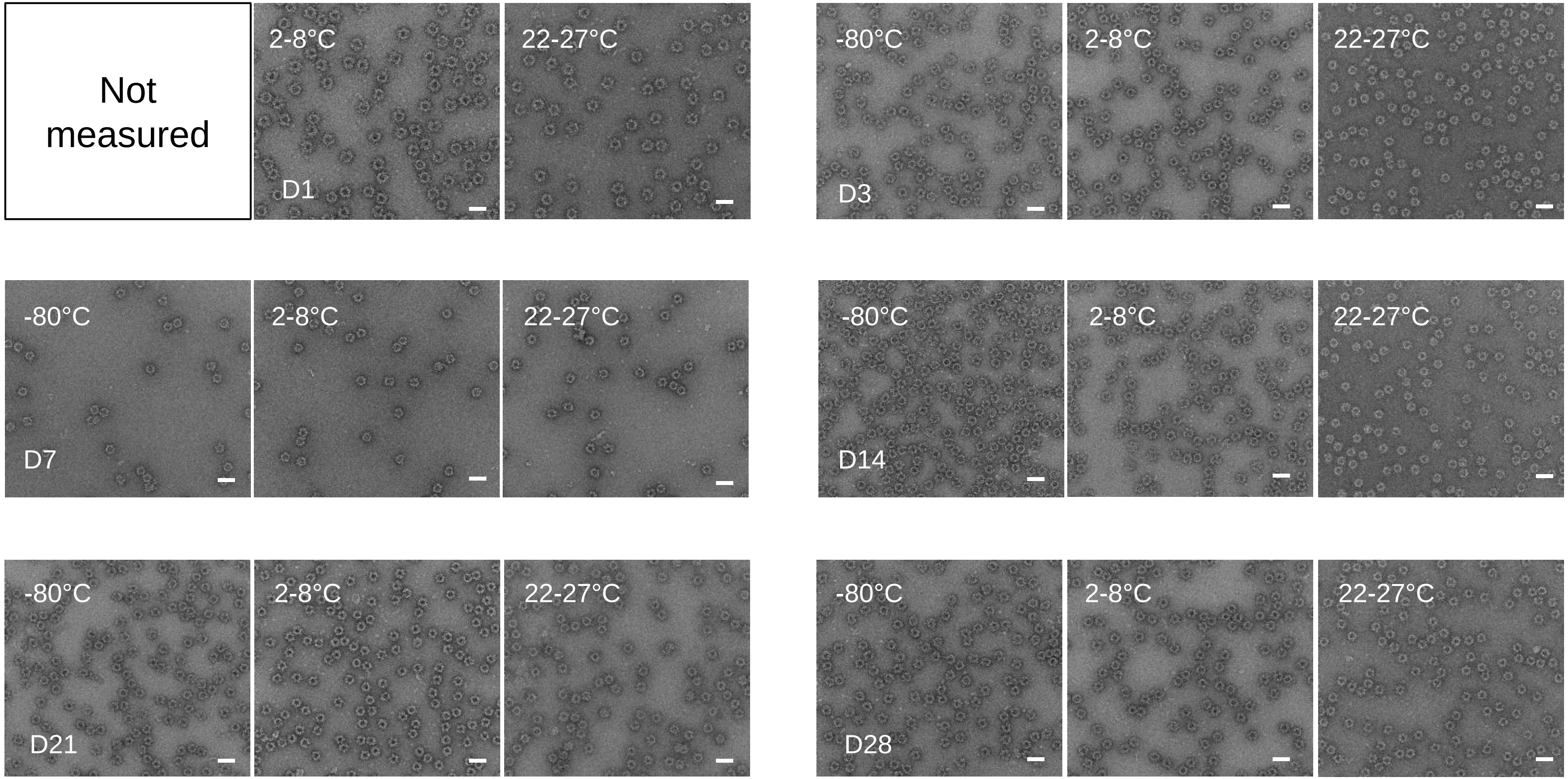

Representative negative stain electron micrographs for each sample at each time point (D, day). Scale bar, 50 nm.

Absorbance at 320/280 nm for RBD-12GS-I53-50 nanoparticle

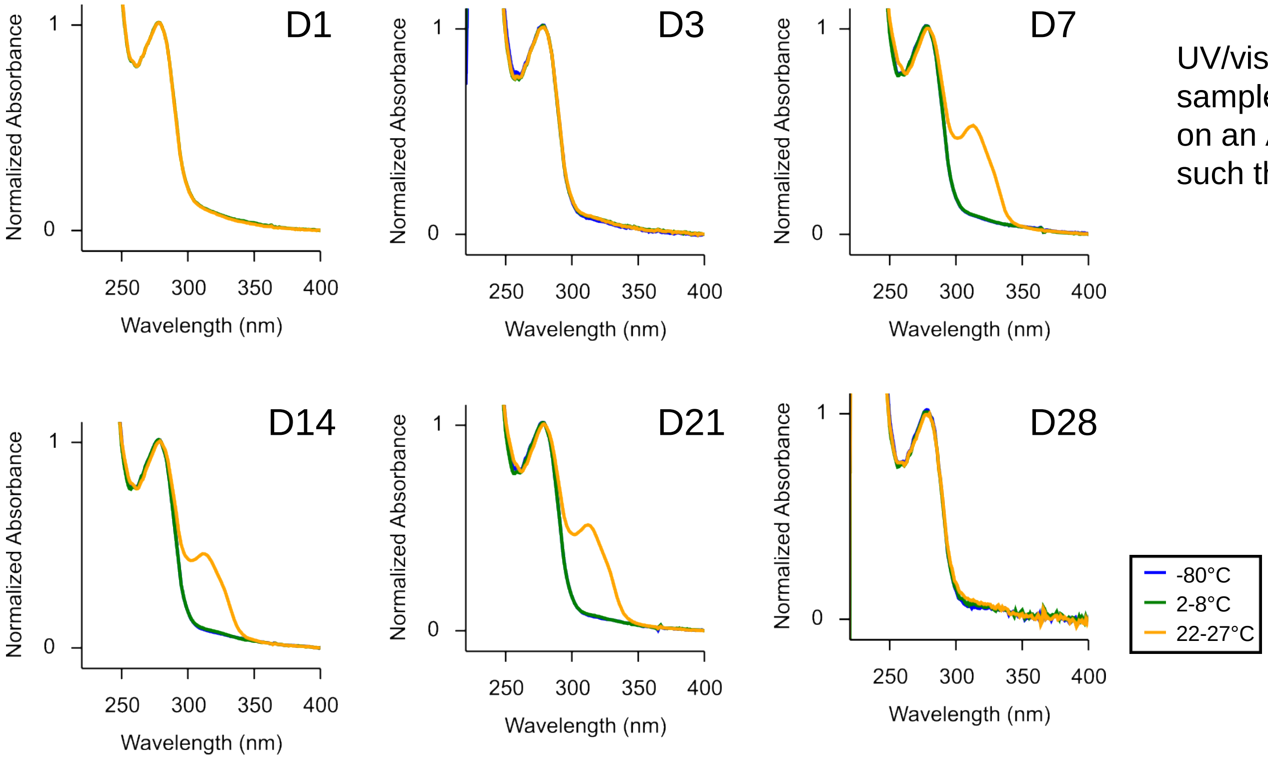

UV/vis absorbance spectra for each sample at each time point were obtained on an Agilent Cary 8454 and normalized such that $A_{280} = 1$ and $A_{400} = 0$.
